# Dietary Habits and Nutrient Intakes Are Associated to Age-Related Central Auditory Processing Disorder in a Cohort From Southern Italy

**DOI:** 10.3389/fnagi.2021.629017

**Published:** 2021-05-06

**Authors:** Luisa Lampignano, Nicola Quaranta, Ilaria Bortone, Sarah Tirelli, Roberta Zupo, Fabio Castellana, Rossella Donghia, Vito Guerra, Chiara Griseta, Pasqua Letizia Pesole, Marcello Chieppa, Giancarlo Logroscino, Madia Lozupone, Anna Maria Cisternino, Giovanni De Pergola, Francesco Panza, Gianluigi Giannelli, Heiner Boeing, Rodolfo Sardone

**Affiliations:** ^1^Unit of Research Methodology and Data Sciences for Population Health, “Salus in Apulia Study” National Institute of Gastroenterology “S. de Bellis” Research Hospital, Bari, Italy; ^2^Otolaryngology Unit, Department of Basic Medical Science, Neuroscience and Sense Organs, University of Bari Aldo Moro, Bari, Italy; ^3^Data Analysis Unit, National Institute of Gastroenterology “S. de Bellis” Research Hospital, Bari, Italy; ^4^Laboratory of Clinical Pathology, National Institute of Gastroenterology “S. de Bellis” Research Hospital, Bari, Italy; ^5^National Institute of Gastroenterology “S. de Bellis” Research Hospital, Bari, Italy; ^6^Center for Neurodegenerative Diseases and the Aging Brain, University of Bari Aldo Moro, Bari, Italy; ^7^Department of Clinical Research in Neurology, “Pia Fondazione Cardinale G. Panico,” Lecce, Italy; ^8^Ambulatory of Clinical Nutrition, National Institute of Gastroenterology “S. de Bellis” Research Hospital, Bari, Italy; ^9^Department of Biomedical Science and Human Oncology, School of Medicine, University of Bari Aldo Moro, Bari, Italy; ^10^German Institute of Human Nutrition Potsdam-Rehbrücke, Nuthetal, Germany

**Keywords:** central auditory processing disorder, ageing, cognition, hearing loss, diet

## Abstract

**Objectives:**

Central auditory processing disorder (CAPD) commonly occurs in older age. However, few studies of a possible link between age-related CAPD and diet in an older population have been conducted. The objective of the present study was to investigate the relationship between eating habits and age-related CAPD in a population >65 years, using cross-sectional and retrospective data obtained in the same population-based study about 12 years ago.

**Methods:**

We selected 734 participants (403 men) from a large population-based study. For age-related CAPD assessment, we used the Synthetic Sentence Identification with Ipsilateral Competitive Message test. Dietary habits were assessed by a Food Frequency Questionnaire. Associations between age-related CAPD and food groups/macro-and micronutrients were explored using adjusted logistic regression models.

**Results:**

Age-related CAPD subjects consumed more dairy (111 vs. 98 g/d), olives and vegetable oil (63 vs. 52 g/d) and spirits (2 vs.1 g/d), and less fruits (536 vs. 651 g/d) in the cross-sectional analysis. Age-related CAPD subjects had a lower intake of potassium, vitamin C, and a higher fat intake. Further analyses identified dietary fiber as being inversely related to age-related CAPD.

**Discussion:**

The present study provided evidence that the dietary hypotheses proposed for explaining the development of cognitive disorders in older age might also hold for age-related CAPD. Further data from other large and prospective population-based studies are needed for confirming these findings.

## Introduction

Central auditory processing disorder (CAPD/ICD-10 H93.25) is a particular diagnostic entity that reflects impaired processing of auditory signals by the central auditory nervous system. The disorder is identifiable in subjects by their inability to understand speech against a competitive message or background noise. It is probably due to an impairment of specific cortical and brain-stem stations deputed to carry out binaural and temporal processing (American Academy of Audiology, 2010). The disorder commonly occurs in older age and is called central presbycusis or age-related CAPD (Sardone et al., 2019). This disorder differs from peripheral presbycusis or peripheral age-related hearing loss (ARHL) in two ways: firstly, because it features a deficit of the nervous system rather than the cochlea, and therefore of the peripheral organ of hearing, and secondly because the diagnosis is based on subjective speech comprehension tests requiring preserved peripheral hearing functions, measured with tonal audiometry (Sardone et al., 2019). Epidemiological studies have observed that age-related CAPD is accompanied by cognitive decline and dementia such as Alzheimer’s disease (AD) (Yuan et al., 2018; Sardone et al., 2019). The cognitive decline associated with ARHL has recently been defined by the provocative term “the cognitive ear” (Sardone et al., 2019), which highlights that hearing signals are not only processed by the ear but also by the auditory cortex and other associative cortical areas (Yuan et al., 2018). The relationship between age-related CAPD and neurodegenerative phenomena may be due to a common underlying microvascular etiology (Sardone et al., 2019), such as a degenerative pathway involving the early formation of neurofibrillary tangles (NFTs) (Sinha et al., 1993). Disorders related to cognitive impairment, and thus age-related CAPD, could be linked to lifestyle, particularly diet (Solfrizzi et al., 2018). Although this critical link has been suggested from different studies (Sardone et al., 2020a; Rodrigo et al., 2021), particularly on peripheral type of age-related hearing loss, there is no evidence of the direction of the association due to the lack of longitudinal or intervention studies on diet and development of CAPD. There are several possible ways in which diet could be connected to late-life cognitive disorders such as age-related CAPD (Beilharz et al., 2015). Two of them are inflammation (Hornedo-Ortega et al., 2018) and a reduction of brain neurotrophism (Ramalho et al., 2018). As regards the first hypothesis, the primary foods to be considered would be fruit and vegetables and associated phytochemicals (McGrattan et al., 2019), while to address the second hypothesis, the primary foods to be considered would be foods containing fats and sugars (Beilharz et al., 2015). Dietary patterns known for their anti-inflammatory effects, such as the Mediterranean diet (MD) (Solfrizzi et al., 2018) and dietary approaches to stop hypertension (DASH), have been found to be neuroprotective. Several nutritional components present in the MD and DASH diets (omega-3 fatty acids, antioxidants, and polyphenols) have been shown to alleviate cognitive impairment-related neuroinflammation (McGrattan et al., 2019). Recently, in the same cohort of this study, we found that plant-based foods, particularly coffee and vegetables, as well as vitamin A sources, were inversely associated to age-related cognitive impairment (Zupo et al., 2021). However, there is a lack of evidence from human trials, and the precise pathways connecting diet to cognitive ability are unknown. More dietary intervention trials are needed to look at diet-related neurological changes from the early stages of cognitive impairment to the end stages (McGrattan et al., 2019). It is also important to note that individuals with late-life cognitive disorders frequently develop changes in eating and dietary habits (Cipriani et al., 2016). The changes may be secondary to cognitive impairment or the result of metabolic or neurochemical abnormalities occurring as part of the dementing process (Cipriani et al., 2016). Studies of a link between age-related CAPD and diet in an older population lack, to the best of our knowledge. In the present study, we investigated how diet may be associated to age-related CAPD, using cross-sectional and retrospective data from a population-based study of community-dwelling older people >65 years in Southern Italy.

## Materials and Methods

### Participants

Participants of the present study were recruited from the electoral rolls of Castellana Grotte, Bari, Southern Italy, within the MICOL studies (*n* = 2472) and the GreatAGE Study (*n* = 2526) (Misciagna et al., 1996; Lozupone et al., 2018b). The prospective Multicenter Italian study on Cholelithiasis from 1985 (MICOL) focused on nutrition and cholelithiasis and colon cancer risk. In 1985, a random sample of 3,500 subjects (2,000 men and 1,500 women) aged ≥30 years was drawn from the electoral roll of Castellana Grotte (17,334 residents at the 1981 Census); 30% of them worked in the agricultural sector and were invited to take part in the study; 2,472 (1,429 men and 1,043 women) of them agreed (70.6% response rate). The cohort was examined several times over the last 35 years. After the initial examination, the study participants were re-invited in 1992–1993 for MICOL2 (M2; 2,159 participants) and in 2005/2006 for MICOL3 (M3; 1,708 participants). M3 included a complete dietary assessment. In 2012, M3-study participants aged 65 years and older were entered into a more extensive population-based study conducted in the same community, the GreatAGE Study, which applied the same dietary assessment as in M3. The GreatAGE study is a population-based study focusing, among other aspects, on the impact of nutrition and age-related sensory impairments as predictors of frailty, neurodegenerative and psychiatric diseases in the elderly (Lozupone et al., 2018a). In 2015, the GreatAGE study was started with an invitation to the previously representative M3 participants. In 2016, it was finally possible to extend the invitation to the whole 65+ population drawn from the administrative national health system data of 2015 (updated to December 31, 2015). The number of residents was 19,675, on December 31, 2015, including 4,537 people aged 65 years or older, including the surviving M3 population.

In the present study, data from the MICOL3 (M3) examination and the GreatAGE Study were used. Recently, the MICOL studies and the GreatAGE Study have been linked to the “Salus in Apulia Study,” a public health initiative funded by the Italian Ministry of Health and Apulia Regional Government and conducted at the IRCCS “S. De Bellis” Research Hospital. In the GreatAGE Study, a hearing assessment was also performed in addition to assessing clinical and lifestyle aspects. We used two different data sets for this study, considering the same subjects (no. 734). The participants underwent only dietary assessment at M3 baseline examination while age-related CAPD was assessed during the GreatAGE Study. The GreatAGE Study participants were assessed cross-sectionally for dietary habits and audiological examination to define age-related CAPD. Data from those participants of the GreatAGE Study who had already participated in the M3 examination (*n* = 734) in order to utilize past data for our investigation. All participants signed informed consent before the examination, and general approval of the studies was obtained from the IRB of the head institution, the National Institute of Gastroenterology and Research Hospital “S. de Bellis” in Castellana Grotte, Italy. The studies were conducted following the 1975 Helsinki Declaration. The present investigation was conducted following the “Standards for Reporting Diagnostic Accuracy Studies” (STARD) guidelines^1^, and the manuscript was organized following the “Strengthening the Reporting of Observational Studies in Epidemiology - Nutritional Epidemiology” (STROBE-nut) guidelines^2^.

### Hearing Assessment

All participants underwent an audiological assessment performed by a qualified audiologist. We collected participants’ tympanometry and stapedial reflexes (Clarinet Plus, Middle Ear Analyzer, Inventis, Italy) to exclude middle and external ear disorders that could induce conductive hearing loss. Lastly, 62 of the 734 eligible subjects were excluded due to the presence of dementia, diseases of the middle ear, or inability to attend the required tests. Following the ICD-10 H93.25 and central presbycusis definition criteria (Jerger et al., 1990; Sardone et al., 2019), age-related CAPD was assessed only in subjects without disabling peripheral ARHL. Disabling peripheral ARHL was evaluated, by pure tone audiometry, as a pure tone average (PTA) threshold greater than 40 dB hearing level (HL) in the better ear according to WHO criteria (Duthey, 2013). Pure tone audiometry was conducted following the Hughson-Westlake method in a soundproof booth with HDR 39 headphones (Sennheiser electronic GmbH & Co. KG, Wedemark, Germany) and a PIANO Audiometer (Inventis SRL, Padova, Italy), The audiometer was calibrated and the examination carried out according to international standards for audiometric testing. To identify age-related CAPD, we used the Italian version of the Synthetic Sentence Identification with Ipsilateral Competitive Message (SSI-ICM) test (Antonelli, 1970), a sensitive, specific measure to define speech intelligibility central patterns. The test consists of administering, for each ear, a primary signal of ten brief sentences against a contextual competition signal (a male talker reading a passage). The test must be administered at a comfortable hearing level for the normal hearing listener (+50 dB sound pressure level over the PTA). The test scoring was expressed as a percentage (0–100%), where 100% is the best performance (Antonelli, 1970). In accordance with Gates et al. (2002, 2011) and Sardone et al. (2019), age-related CAPD was considered present when the patient scored <50% in at least one ear. Normal hearing subjects and peripheral ARHL (PTA lower than 40 dB HL) were labeled non-age-related CAPD subjects in the comparison and association analyses.

### Dietary and Clinical Assessment

Diet was assessed with the same food frequency method applied in the previous examinations. The self-administered food frequency questionnaire (FFQ) was structured in eleven sections, including foods of similar characteristics: grains, meat, fish, milk and dairy products, vegetables, legumes, fruits, miscellaneous foods, water and alcoholic beverages, olive oil and other edible fats, coffee/sugar and salt. In a further step, the FFQ was validated against dietary records, and the results were reviewed to adapt the questionnaire to our population (Leoci et al., 1993). In the final questionnaire, 85 food items were considered to best reflect the regional diet, together with some questions about the use of edible fats. The 85 food items in the FFQ and the questions about the use of fat were regrouped and further summarized under 30 food groups. One food group (edible cooking fats) could not be quantified and was not used in the present study because on this food group, the FFQ refers only to the frequency and not the quantity of intakes. Total energy and intake of water, protein, fats (also divided into saturated fat, monounsaturated fat, polyunsaturated fat and cholesterol), carbohydrates, fibers, alcohol, sodium, potassium, iron, calcium, phosphorus, thiamine, riboflavin, niacin, and vitamin A and vitamin C were calculated from food intake data using the Italian food composition table (Carnovale and Miuccio, 1987).

The clinical examination included an interview and a questionnaire which also covered socioeconomic and lifestyle variables such as years of education and smoking habit. The education variable was classified based on the Italian national education system. The lowest level, <6 years, reflected primary school education, the middle level, 6–8 years, reflected lower secondary school education, and the highest level, >8 years, reflected upper secondary school/high school education and university education. Smoking habit was assessed with the single question “Are you a current smoker?”, scored yes or no.

Height and weight measurements were performed using a Seca 220 altimeter and a Seca 711 scale. Body mass index (BMI) was calculated as kg/m^2^. Multimorbidity status was defined as the presence of two or more chronic diseases, among the following conditions: diabetes, hypertension, peptic ulcer, cholangiolithiasis, myocardial infarction, hepatic cirrhosis or other liver diseases, inflammatory bowel diseases, major infectious diseases, leukemia or other blood chronic diseases, viral hepatitis, and AIDS (World Health Organization (WHO), 2016).

### Statistical Analysis

Participants characteristics are reported as mean and standard deviation (M ± SD) for continuous variables and as frequencies and percentages (%) for categorical variables. The study population was examined regarding lifestyle, clinical parameters, and diet in two different periods, retrospectively at the M3 examination about 12 years ago and cross-sectionally together with the hearing assessment (GreatAGE Study). For each examination period, the subjects were subdivided into two categories: age-related CAPD (*Yes/No*). The means for sociodemographic, anthropometric, clinical, and clinical-chemical characteristics, as well as the intake of foods, food groups and nutrients, were adjusted for age, sex, smoking habit, education, BMI, diabetes mellitus, and anti-hypertensive and statins drug use. The respective adjustments for each variable are reported in the notes under the tables. The *p*-values for the association between age-related CAPD and single factors were derived from adjusted logistic regression models using the same covariates set for the adjusted means. The intake of foods, food groups and nutrients, was calculated as daily consumption in g/d. Nutrient intakes were energy-adjusted with the residual method applying regression models, using total energy intake as the independent variable and nutrient intake as the dependent variable (Willett and Stampfer, 1986). A *p*-value of <0.05 was considered significant. For formal analyses, STATA 16.0, StataCorp. 2019 software was used (*Stata Statistical Software: Release 16*. College Station, TX, United States: StataCorp LLC).

## Results

In the participants examined at M3 and GreatAGE Study, the male sex was slightly predominant, accounting for 55% (Table 1). Among the 672 participants with audiological assessment, age-related CAPD was diagnosed in 199 subjects at GreatAGE Study. Age-related CAPD participants were older and had lower education levels than non-age-related CAPD participants. Participants with and without age-related CAPD did not differ as regards the other variables. Moreover, there were no significant differences in the clinical and lifestyle variables nor the metabolic profile between groups at the examinations over time (M3 to GreatAGE) (Table 1).

**TABLE 1:** Sociodemographic and clinical characteristics of the population of the GreatAGE Study examination (M4) and about 12 years back in time of the MICOL3 examination (M3) (*n* = 734, non-age-related CAPD *n* = 473, age-related CAPD *n* = 199, missing *n* = 62).

Variables*	GreatAGE Study (M4) (2012–2018)			MICOL3 (M3) (2005–2006)		
	Age-related CAPD			Age-related CAPD		
	No (≥50%) *n* = 473	Yes (<50%) *n* = 199	Effect Size^Ψ^ (95% C.I.)	No (≥50%) *n* = 473	Yes (<50%) *n* = 199	Effect Size^Ψ^ (95% C.I.)
Sex, males (%)	283 (59.83)	119 (59.80)	0.96 (−0.08 to 0.08)	283 (59.83)	119 (59.80)	0.99 (−0.08 to 0.08)
Age (years)	71.29 ± 5.55	76.83 ± 5.74	−0.99 (−1.16 to −0.81)	63.77 ± 5.57	69.30 ± 5.93	−0.97 (−1.14 to −0.80)
Smoke (%)^(a)^	52 (11.08)	12 (6.12)	0.03 (−0.09 to −0.01)	65 (13.95)	19 (9.09)	0.10 (−0.09 to 0.01)
Education (%)^(b)^						
Low	223 (47.52)	139 (70.14)	−0.46 (−0.63 to −0.30)	272 (58.09)	171 (86.34)	−0.62 (−0.79 to −0.45)
Medium	124 (26.35)	33 (16.68)	0.23 (0.06 to 0.40)	109 (23.31)	19 (9.61)	0.35 (0.18 to 0.52)
High	123 (26.13)	26 (13.17)	0.31 (0.15 to 0.48)	87 (18.60)	8 (4.05)	0.42 (0.25 to 0.59)
BMI (kg/m^2^)	28.99 ± 4.81	29.15 ± 5.54	−0.03 (−0.20 to 0.13)	29.60 ± 4.72	29.97 ± 5.23	−0.07 (−0.24 to 0.09)

**CAPD, central auditory processing disorder; BMI, body mass index. ^Ψ^Hedges’s Effect Size; (95% C.I.), Confidential Interval at 95%.*^∗^Reported as Mean and Standard Deviation (M ± SD). Mean and logistic regression model adjusted for: (a) age, gender, and education; (b) age and gender. MICOL 3 and GreatAGE Study.*

Table 2 shows the food intakes of subjects with and without age-related CAPD, with the multivariate adjusted odds-ratio of difference for the cross-sectional analysis and the period of about 12 years before the audiological assessment. Differences between subjects with and without age-related CAPD appeared to dominate the results of the cross-sectional analysis. Twelve years before the audiological evaluation, we observed a difference only for grains, for which a higher intake by about 20 g/d had been recorded in subjects with age-related CAPD. In the cross-sectional analyses, subjects with age-related CAPD ate more dairy foods (110 g/d in the age-related CAPD group vs. 98 g/d in the non-age-related CAPD group), olives and vegetable oil (63 g/d in the age-related CAPD group vs. 52 g/d in the non-age-related CAPD group), and fewer fruits (536 g/d in the age-related CAPD group vs. 651 g/d in the non-age-related CAPD group). Also, age-related CAPD subjects drank more spirits (2 g/d in the age-related CAPD group vs. 1 g/d in the non-age-related CAPD group). There were no other significant differences in food intake between these groups, not even for those mainly considered to have anti-inflammatory properties (vegetable foods, nuts, or legumes), apart from fruits.

**TABLE 2:** Dietary characteristics of the population of the GreatAGE Study examination and about 12 years back in time of the MICOL3 examination (M3) (*n* = 734, non-age-related CAPD *n* = 473, age-related CAPD *n* = 199, missing *n* = 62).

	GreatAGE study *(2012–2018)*				MICOL 3 *(2005–2006)*		
	Age-related CAPD				Age-related CAPD		
Variables*	No (≥50%)° *n* = 473	Yes (<50%)° *n* = 199	OR (95% C.I.)^#^		No (≥50%)° *n* = 473	Yes (<50%)° *n* = 199	OR (95% C.I.)^#^
Food Groups^(c)^							
Dairy	98.16 ± 9.98	110.64 ± 29.84	1.002 (1.001 to 1.004)	↑	95.09 ± 11.63	96.92 ± 12.04	1.001 (0.999 to 1.003)
Low fat dairy	100.98 ± 14.49	94.34 ± 28.71	0.998 (0.996 to 1.000)		92.42 ± 14.35	95.49 ± 25.05	0.999 (0.998 to 1.002)
Eggs	7.30 ± 0.71	7.56 ± 1.67	1.005 (0.981 to 1.029)		7.51 ± 0.91	7.48 ± 0.84	1.010 (0.988 to 1.032)
White meat	25.38 ± 4.74	25.75 ± 3.84	1.001 (0.995 to 1.008)		23.50 ± 3.85	23.11 ± 3.69	0.998 (0.989 to 1.007)
Red meat	24.28 ± 5.07	25.67 ± 7.04	1.003 (0.994 to 1.012)		31.64 ± 6.55	28.33 ± 8.12	0.993 (0.984 to 1.002)
Processed meat	14.78 ± 2.62	15.33 ± 6.59	1.013 (0.999 to 1.026)		17.01 ± 5.57	15.17 ± 5.95	1.003 (0.991 to 1.015)
Fish	26.70 ± 3.43	24.34 ± 4.88	1.001 (0.993 to 1.010)		26.76 ± 6.08	24.87 ± 3.53	0.999 (0.991 to 1.008)
Seafood/shellfish	10.27 ± 1.33	9.70 ± 1.51	1.001 (0.987 to 1.015)		11.57 ± 1.92	11.32 ± 3.78	0.998 (0.982 to 1.014)
Leafy vegetables	57.81 ± 5.70	65.16 ± 13.16	1.001 (0.998 to 1.004)		62.81 ± 9.97	68.44 ± 14.36	1.000 (0.997 to 1.003)
Fruiting vegetables	96.64 ± 6.19	97.16 ± 8.03	0.999 (0.998 to 1.002)		97.98 ± 10.62	109.23 ± 18.13	1.001 (0.999 to 1.004)
Root vegetables	10.72 ± 4.19	13.78 ± 4.95	0.999 (0.993 to 1.006)		8.35 ± 2.46	7.58 ± 0.97	0.999 (0.999 to 1.009)
Other vegetables	84.60 ± 11.86	88.92 ± 16.90	1.001 (0.998 to 1.003)		77.16 ± 9.60	89.34 ± 18.91	1.001 (0.999 to 1.004)
Legumes	36.28 ± 2.75	40.35 ± 6.00	1.002 (0.995 to 1.010)		37.88 ± 4.30	41.93 ± 4.25	1.002 (0.995 to 1.008)
Potatoes	13.87 ± 2.68	16.32 ± 3.15	1.000 (0.992 to 1.008)		14.85 ± 1.61	16.18 ± 3.91	1.000 (0.990 to 1.011)

	**GreatAGE study *(2012–2018)***				**MICOL 3 *(2005–2006)***		
	**Age-related CAPD**				**Age-related CAPD**		
Variables*	**No (≥50%)° *n* = 473**	**Yes (<50%)° *n* = 199**	**OR (95% C.I.)^#^**		**No (≥50%)° *n* = 473**	**Yes (<50%)° *n* = 199**	**OR (95% C.I.)^#^**

Food groups							
Fruits	651.39 ± 98.14	535.74 ± 80.33	0.999 (0.998 to 0.999)	↓	671.56 ± 96.32	656.20 ± 68.87	1.000 (0.999 to 1.000)
Nuts	5.75 ± 1.66	4.98 ± 1.18	0.997 (0.982 to 1.012)		3.24 ± 0.68	4.34 ± 2.08	1.007 (0.988 to 1.025)
Grains	167.28 ± 25.07	170.22 ± 30.80	1.001 (0.999 to 1.002)		196.25 ± 37.48	215.22 ± 35.15	1.002 (1.000 to 1.003)
Olives and vegetable Oil	51.65 ± 2.85	63.40 ± 15.59	1.005 (1.001 to 1.009)	↑	48.06 ± 5.16	50.94 ± 5.11	0.998 (0.991 to 1.005)
Sweets	23.13 ± 4.69	20.53 ± 5.23	0.998 (0.992 to 1.005)		19.58 ± 3.45	19.87 ± 4.34	1.003 (0.995 to 1.010)
Sugary	9.59 ± 1.23	12.00 ± 2.78	1.012 (0.996 to 1.029)		11.71 ± 1.93	15.06 ± 4.46	1.015 (0.999 to 1.032)
Juices	6.98 ± 1.62	6.29 ± 4.05	1.002 (0.991 to 1.011)		12.23 ± 8.60	14.03 ± 12.62	1.002 (0.999 to 1.005)
Caloric drinks	6.66 ± 6.70	6.58 ± 6.41	1.002 (0.996 to 1.007)		11.28 ± 6.50	10.70 ± 11.84	1.001 (0.997 to 1.005)
Ready to eat dish	30.82 ± 4.18	28.09 ± 4.77	1.000 (0.994 to 1.007)		36.29 ± 6.67	32.41 ± 8.11	1.001 (0.996 to 1.005)
Coffee	50.26 ± 8.94	40.78 ± 6.93	0.994 (0.986 to 1.001)		53.42 ± 10.74	44.91 ± 9.84	0.997 (0.999 to 1.004)
Wine	143.95 ± 78.23	141.35 ± 85.64	0.999 (0.999 to 1.001)		175.87 ± 105.35	188.12 ± 116.92	1.000 (0.999 to 1.001)
Beer	19.99 ± 15.46	20.46 ± 28.35	1.001 (0.998 to 1.003)		27.98 ± 24.09	36.73 ± 36.12	1.001 (0.999 to 1.003)
Spirits	1.06 ± 0.65	1.77 ± 2.32	1.051 (1.001 to 1.104)		1.53 ± 1.26	2.37 ± 3.43	1.024 (0.996 to 1.052)
Water	667.46 ± 23.87	658.68 ± 27.05	0.999 (0.999 to 1.001)		612.33 ± 51.45	625.43 ± 66.72	1.000 (0.999 to 1.001)

**CAPD, central auditory processing disorder. ^∗^Reported as Mean and Standard Deviation (M ± SD). # OR, Odds Ratio at 95% Confidential Interval in the regression model (≥50% vs. <50%) °50% of Synthetic Sentence Identification with Ipsilateral Competitive Message test in background noise. Mean and logistic regression model adjusted for: (c) age, gender, smoking, education, and body mass index. MICOL 3 and GreatAGE Study: All Foods Index and Food Groups were calculated on quantity daily consumption; ↑ increased risk, ↓decreased risk*.*

The differences in food intake could result in further differences in nutrient intake, calculated from the food intake. Thus, we also explored differences in the consumption of micro and macronutrients. Table 3 shows the consumption of micronutrients in subjects with and without age-related CAPD, primarily supporting the inflammatory hypothesis linked to diet. We found less intake of vitamin C and potassium, both high in fruit, in age-related CAPD subjects compared to non-age-related CAPD subjects. However, the groups did not differ regarding other micronutrients included in the inflammatory hypothesis linked to diet. Energy intake was slightly different between the groups, being slightly higher in the age-related CAPD subjects, but this difference was not significant (Table 4). Thus, both the macronutrient and micronutrients intakes were adjusted for energy. The analyses regarding macronutrients show a good fit with the neurotrophic hypothesis linked to diet. The energy-adjusted intake of fats, particularly saturated and monounsaturated fatty acids, was higher among the age-related CAPD subjects compared to the non-age-related CAPD subjects, accompanied by a lesser carbohydrate intake. The latter seems logical when assuming energy equilibrium. An additional finding regards fibers, showing a lesser consumption in age-related CAPD subjects. No difference in macro and micronutrient intake was found at the M3 examination, also relating to carbohydrates., as shown by the results for grains. A similar finding was observed for alcohol intake, which was not different between the groups despite the slightly higher consumption of spirits in the age-related CAPD group.

**TABLE 3:** Micronutrient intake characteristics of the population at baseline (M3) and follow-up (GreatAGE study) (*n* = 734, non-age-related CAPD *n* = 473, age-related CAPD *n* = 199, missing *n* = 62).

	GreatAGE study (2012–2018)			MICOL 3 (2005–2006)			
	Age-related CAPD				Age-related CAPD		
Variables*	No (≥50%)° *n* = 473	Yes (<50%)° *n* = 199	OR (95% C.I.)^#^		No (≥50%)° *n* = 473	Yes (<50%)° *n* = 199	OR (95% C.I.)^#^
Micronutrients							
Sodium	1529.60 ± 73.85	1584.00 ± 119.88	1.000 (0.999 to 1.000)		1662.40 ± 86.91	1657.72 ± 88.89	1.000 (0.999 to 1.000)
Potassium	3459.50 ± 183.59	3186.55 ± 199.87	0.999 (0.998 to 0.999)	↓	3608.39 ± 267.91	3523.73 ± 205.38	0.999 (0.999 to 1.000)
Iron	11.37 ± 0.57	11.00 ± 0.95	0.951 (0.899 to 1.005)		12.19 ± 0.65	12.13 ± 0.84	0.985 (0.920 to 1.054)
Calcium	866.56 ± 83.66	883.38 ± 101.44	0.999 (0.999 to 1.000)		884.04 ± 90.11	914.77 ± 85.19	1.000 (0.999 to 1.001)
Phosphorus	1140.24 ± 23.23	1138.44 ± 89.28	0.999 (0.999 to 1.000)		1208.47 ± 42.29	1224.81 ± 61.00	1.000 (0.999 to 1.001)
Thiamine	0.84 ± 0.04	0.81 ± 0.04	0.557 (0.264 to 1.175)		0.88 ± 0.04	0.86 ± 0.05	0.860 (0.380 to 1.944)
Riboflavin	1.43 ± 0.05	1.41 ± 0.09	0.898 (0.620 to 1.301)		1.47 ± 0.08	1.47 ± 0.11	1.017 (0.643 to 1.607)
Niacin	1.43 ± 0.05	1.41 ± 0.09	0.898 (0.620 to 1.301)		1.47 ± 0.08	1.47 ± 0.11	1.017 (0.643 to 1.607)
Vitamin A	1186.15 ± 138.63	1163.19 ± 115.01	0.999 (0.999 to 1.000)		1158.90 ± 97.16	1101.90 ± 128.14	0.999 (0.999 to 1.000)
Vitamin C	189.33 ± 16.16	171.21 ± 21.84	0.998 (0.996 to 0.999)	↓	188.76 ± 24.39	182.88 ± 22.41	0.999 (0.998 to 1.001)

**CAPD, central auditory processing disorder.*^∗^Reported as Mean and Standard Deviation (M ± SD). # Odds Ratio at 95% Confidential Interval in the regression model (≥50% vs. <50%). Mean and logistic regression model adjusted for: (c) age, gender, smoking, education, and BMI; ↓ decreased risk. *MICOL 3 and GreatAGE Study: All Foods Index and Food Groups were calculated on quantity daily consumption*.*

**TABLE 4:** Macronutrient intake characteristics of the population at baseline (M3) and follow-up (GreatAGE study) (*n* = 734, non-age-related CAPD *n* = 473, age-related CAPD *n* = 199, missing *n* = 62).

	GreatAGE study (2012–2018)			MICOL 3 (2005–2006)			
	Age-related CAPD				Age-related CAPD		
Variables *	No (≥50%)° *n* = 473	Yes (<50%)° *n* = 199	OR (95% C.I.)^#^		No (≥50%)° *n* = 473	Yes (<50%)° *n* = 199	OR (95% C.I.)^#^
Macronutrients^(c)^							
Water	2003.54 ± 108.74	1904.75 ± 157.89	0.999 (0.999 to 1.000)		2018.74 ± 147.68	2063.06 ± 284.87	0.999 (0.999 to 1.000)
Protein	67.16 ± 2.71	67.28 ± 3.47	1.001 (0.992 to 1.011)		72.30 ± 3.82	71.54 ± 3.31	1.002 (0.999 to 1.014)
Fats	82.46 ± 6.63	90.01 ± 12.23	1.008 (1.002 to 1.014)	↑	80.33 ± 5.14	80.82 ± 5.10	0.995 (0.986 to 1.004)
Carbohydrate	408.95 ± 23.27	375.43 ± 40.33	0.998 (0.996 to 0.999)	↓	275.12 ± 8.97	273.95 ± 15.09	1.000 (0.999 to 1.002)
Fibers	63.88 ± 5.80	58.16 ± 5.93	0.990 (0.984 to 0.997)	↓	26.50 ± 2.56	26.72 ± 1.83	1.001 (0.994 to 1.008)
Saturated fat	21.39 ± 2.03	23.02 ± 2.56	1.032 (1.006 to 1.060)	↑	21.52 ± 1.45	21.65 ± 1.25	1.000 (0.974 to 1.028)
Monounsaturated fat	43.43 ± 3.83	48.80 ± 8.78	1.008 (1.001 to 1.015)	↑	41.73 ± 3.66	42.32 ± 3.37	0.990 (0.977 to 1.003)
Polyunsaturated Fat	9.06 ± 0.64	9.37 ± 1.21	1.045 (0.983 to 1.108)		8.65 ± 0.53	8.74 ± 0.51	0.955 (0.879 to 1.038)
Cholesterol	187.27 ± 15.49	186.14 ± 16.36	1.000 (0.998 to 1.002)		193.16 ± 15.87	184.14 ± 12.88	0.999 (0.997 to 1.002)
Alcohol	15.80 ± 7.81	15.17 ± 9.16	0.996 (0.984 to 1.008)		19.68 ± 9.25	20.14 ± 9.85	0.999 (0.999 to 1.008)
Kcal	2055.02 ± 116.29	2120.27 ± 249.06	1.000 (0.999 to 1.000)		2161.70 ± 232.63	2262.26 ± 321.83	1.000 (0.999 to 1.000)
KJ	8602.17 ± 486.59	8875.22 ± 1042.63	1.000 (0.999 to 1.000)		9048.53 ± 973.53	9469.57 ± 1347.27	1.000 (0.999 to 1.000)

**CAPD, central auditory processing disorder; Kcal, kilocalorie; KJ, kilojoule.*^∗^Reported as: Mean and Standard Deviation (M ± SD) # OR, Odds Ratio at 95% Confidential Interval in regression model (≥50% vs. <50%). *Mean and logistic regression model adjusted for: (c) age, gender, smoking, education, and body mass index; ↑ increased risk, ↓ decreased risk. MICOL 3 and GreatAGE Study: All Foods Index and Food Groups were calculated on quantity daily consumption.**

## Discussion

### Summary of Main Findings

In the present study on diet and age-related CAPD, dairy foods, as sources of fat, and vegetable oils, including olives and fats (particularly saturated and monounsaturated fatty acids) as primary macronutrients, were positively linked to age-related CAPD. On the other hand, fruits as well as potassium and vitamin C, as micronutrients, were inversely associated with age-related CAPD. Furthermore, we found a positive association of age-related CAPD with the intake of spirits, but not of alcohol in general. The findings regarding carbohydrates as macronutrients could mirror the results relating to fat consumption. The retrospective dietary analyses did not reveal much support for dietary factors examined at baseline that may contribute to the subsequent development of age-related CAPD.

### Comparison With Other Studies

In agreement with other similar findings, we could confirm that CAPD is age-related and particularly affected subjects with fewer years of education (Sardone et al., 2020a). The result of the relation to age in this study population has already been shown, suggesting that age-related CAPD was highly frequent in this population, accounting for 12%, and is related to dementia and mild cognitive impairment (Sardone et al., 2020a). Our finding regarding lower education in age-related CAPD is consistent with other recent analyses (Sardone et al., 2020a), and also in line with the concept of age-related CAPD and cognitive impairment as two sides of the same coin. Educational level has recently been linked with general auditory processing skills (Sardone et al., 2020a). Probably, the better performance of individuals with a higher educational level may be due to environmental enrichment, which could be linked to a greater number of synapses and vascularisation, and, therefore, to changes in the brain structure occurring early in life (Rogowsky et al., 2013). Moreover, the present study could not relate age-related CAPD with clinical chemistry and metabolic biomarkers. The prevalence of diabetes mellitus was increased in age-related CAPD subjects, but not to a significant extent.

Since this is one of the first studies to focus on diet and age-related CAPD, we could only derive our hypotheses about how foods could interact with age-related CAPD from findings on the links among dietary factors and late-life cognitive disorders, assuming that age-related CAPD and cognitive impairment may be associated (Sardone et al., 2020a). One of our hypotheses, which was confirmed by the present data, concerns the role of dietary fat concerning reduced neurotrophism. Dietary fats intake has been found to be related to impaired cognition in several studies (Beilharz et al., 2015). In the Italian Longitudinal Study on Aging, Solfrizzi et al. (2006) found a positive association between the intake of monounsaturated and polyunsaturated fatty acids and low scores on cognitive testing in non-demented older subjects. In support of the present finding on dairy foods, a cohort study found that the group consuming full-cream milk regularly showed a significant decrease in successful mental health aging compared to the group rarely consuming this food (Almeida et al., 2006). Other recent experimental studies showed microglial cell activation increases in response to a high-fat diet, and this phenomenon was linked to impoverished cognitive functions (Baufeld et al., 2016). A high-fat diet specifically stimulates endogenous microglia in the hypothalamus, and that the microglial response is not exclusively pro-inflammatory. Long-term exposure to this particular kind of diet results in an altered microglia profile represented by downregulation of microglia-specific genes involved in sensing microenvironmental alterations, supposedly serving to counterbalance earlier pro-inflammatory changes. This type of response appears to be a typical reaction of microglia to chronic diseases (Baufeld et al., 2016). There is also the support of the concept that a high fats consumption may alter negatively neurotrophism during aging (Norden and Godbout, 2013; Smith, 2013). It is well known that adipose tissue could be considered an endocrine organ, producing multiple signaling proteins designated adipokines (Trayhurn et al., 2008). Adipose tissue and fat in general could modulate the production of two of the most important neurotrophins active in the brain and involved in several neurodegeneration processes. Those adipokines are the nerve growth factor (NGF) and adipose tissue-derived brain-derived neurotrophic factor (BDNF) (Sornelli et al., 2007). In addition to their stimulatory action on neuronal growth and survival, neurotrophins also act on several other cell types, including immune cells (Aloe et al., 2001) and pancreatic β cells (Yamanaka et al., 2006). Moreover, NGF and BDNF were also known as metabokines (Sornelli et al., 2007) for the role in metabotrophic effects on glucose, lipid and energy homeostasis (Tore et al., 2007). Several recent studies found altered levels of fat-derived neurotrophins in pathological conditions due to metabolic, cognitive or behavioral disorders (Allen et al., 2011). Moreover, high-fat consumption has been associated with reductions of BDNF and impaired neurogenesis in murine models (Park et al., 2010). According to Ramalho et al. (2018), the median eminence/spinal fluid interface is affected at the functional and structural levels after introducing a high-fat diet. BDNF supplies early protection against damage, which is lost upon a continued consumption of large amounts of dietary fats. This is of particular interest because of the role of BDNF as an essential mediator of neurotrophism, both in the lower parts of the auditory cortex, involved in age-related CAPD neuropathology and in the hippocampus (the most crucial driver of memory) (Chumak et al., 2016). Adult neurogenesis is located in only two regions of the brain: the hippocampus and the prefrontal cortex. Adult hippocampal neurogenesis has been shown to be improved by exercise, enriched environments, and caloric restriction (Das and Basu, 2008), while it has been shown to be reduced by stress, low-grade inflammation, oxidative stress, and aging (Dias et al., 2012). Interestingly, lower intakes of nutrient-dense foods and higher intakes of unhealthy foods were also correlated with smaller left hippocampal volumes in a cohort study of community-based older adults (Jacka et al., 2015).

A further important finding of the present study was that higher consumption of fruit, known to have antioxidant properties, was associated with a better central auditory function. In other animal studies, it was observed that a higher intake of vitamin C was related to better auditory functions (Alvarado et al., 2018). Some population-based studies suggested that the antioxidant vitamin E and vitamin C in the diet have also been associated with a reduced risk of dementia (Engelhart et al., 2002). Moreover, other population-based studies have demonstrated that dietary levels of fruit intake and vitamin C are inversely related to levels of C reactive protein (CRP), an inflammatory marker (Wannamethee et al., 2006) associated with age-related chronic diseases (Hulsegge et al., 2016), cognitive impairment (Gu et al., 2018; Lin et al., 2018), and frailty (Soysal et al., 2016). Besides, experimental and clinical-based evidence suggested that an increased intake of potassium, highly concentrated in some fruits (e.g., melon, apricots, and kiwis), could help to prevent health disorders such as hypertension or possibly prevent or delay the onset of cognition-related conditions such as AD (Cisternas et al., 2015; Zupo et al., 2019). Interestingly, in a previous study on the same population, we found that subjects with ARHL, a chronic disease affecting peripheral hearing, consumed more pro-inflammatory foods and a lower amount of vitamin A (another molecule with a well-known antioxidant power) than subjects without ARHL (Sardone et al., 2020b).

Despite we assume the link between fats and carbohydrates under the assumption of energy equilibrium, the finding regarding fiber could be of interest, mainly since the retrospective analysis found an inverse relation with grain intake. In a large cohort of older adults, a higher glycemic index (GI) of foods consumption was associated with an increased prevalence of ARHL (Gopinath et al., 2010). The link between GI, diabetes and potentially age-related CAPD should be further explored, as well as the link with antioxidants.

### CAPD and Cognitive Decline

A number of epidemiological evidences suggesting a link between central auditory dysfunction and cognitive decline, the causal mechanisms underlying this association are substantially unknown (Sardone et al., 2019, 2020a). Important neuropathological research supported the hypothesis that age-related CAPD may result from a degenerative pathway other than cognitive decline observed in AD, showing that brain amyloid-β, believed to be the initial event characterizing AD, was uncommon in central auditory pathways early in the clinical course of the disease (Sinha et al., 1993). By contrast, there was early formation of neurofibrillary tangles (NFTs), mainly consisting of hyperphosphorylated tau protein, suggesting that neurodegeneration in the auditory cortex may be an ongoing process the AD course (Sinha et al., 1993). These seminal findings and the neurobiological plausibility of this relationship have recently been confirmed by suggestive neuropathological results showing an association of clinician-reported ARHL with the highest Braak stage, suggesting an increased NFT burden in cognitively unimpaired/hearing impaired subjects (Brenowitz et al., 2020; Lozupone et al., 2020). In particular, the most severe Braak stage involves central auditory processing core areas, that is, the superior temporal gyrus and the primary auditory cortex (Brenowitz et al., 2020; Lozupone et al., 2020). These findings increase the attention on age-related CAPD as a cognitive-hearing impairment. Furthermore, the relationship between age-related CAPD and NFT-based neurodegenerative phenomena could lay in a shared underlying microvascular etiology. Given that the diagnosis of age-related CAPD is much simpler than the clinical diagnosis of dementia—which needs comprehensive neuropsychological and imaging features—central auditory dysfunction could be an important element to be monitored by clinicians, particularly geriatricians. However, a limitation of monitoring age-related CAPD is that its diagnosis is based only on subjective indicators of speech perception, and therefore may require accessory objective biomarkers able to confirm its presence. Given the neurovascular implications of central auditory dysfunction, one of the methods could be the use of retinal vascular biomarkers, which have been found cross-sectionally associated with age-related CAPD in a recent study involving our cohort (Sardone et al., 2020c).

### Strengths and Limitations

The strengths of the present study included its well-defined population-based sample, the standardized, clinically and instrumentally based audiometric assessments to measure age-related CAPD, and the use of a validated FFQ to collect dietary information. However, some limitations must be considered. There is a potential for misclassification regarding dietary intake since the information was collected by self-report, which is liable to recall bias even though the FFQ was designed according to common principles that attempt to minimize this type of error. Besides, it was impossible to consider important covariates, such as medication or economic conditions, because data were not available. Furthermore, the cross-sectional data do not reveal a clear directionality of the association. Since the retrospective analyses were primarily negative in terms of associations, we cannot exclude the possibility that age-related CAPD changed dietary behavior (Cipriani et al., 2016). Moreover, we have not measured plasma levels of CRP as well as other inflammatory cytokines to support our thesis about increased inflammation, also considering that the assays of cytokines could be severely affected by the time because the kinetics of that molecules could vary in a short period in the same subject.

## Conclusion

The present results showing how dietary intakes and age-related CAPD could be linked and confirming dietary hypotheses explaining the development of late-life cognitive disorders may be hypothesis-generating findings in line with a proposed link between central auditory function and cognitive impairment. Further research into this topic seems warranted and could result in a more solid knowledge of this issue. Together with better screening of age-related CAPD in older people, mainly when other cognitive disorders are present, these findings could yield better prospects for the prevention and treatment of this and hopefully also other psychoacoustic disorders.

## Data Availability Statement

The raw data supporting the conclusions of this article will be made available by the authors, without undue reservation.

## Ethics Statement

The studies involving human participants were reviewed and approved by IRB Istituto Tumori “Giovanni Paolo II” IRCCS. The patients/participants provided their written informed consent to participate in this study.

## Author Contributions

HB, NQ, and RS: study conception and design. IB, ST, RZ, FC, CG, AC, PP, and MC: acquisition of data. RD and VG: analysis and interpretation of data. LL: drafting of manuscript. HB, FP, ML, GD, and GG: critical revision. All authors contributed to the article and approved the submitted version.

## Conflict of Interest

The authors declare that the research was conducted in the absence of any commercial or financial relationships that could be construed as a potential conflict of interest.

## Footnotes

1 http://www.stard-statement.org/

2 http://www.strobe-nut.org/
